# Aging, Pensions and Long-term Care: What, Why, Who, How? Comment on "Financing Long-term Care: Lessons From Japan"

**DOI:** 10.15171/ijhpm.2019.117

**Published:** 2019-11-25

**Authors:** Kieke Okma, Michael K. Gusmano

**Affiliations:** ^1^Independent Researcher.; ^2^School of Public Health, Rutgers University, New Brunswick, NJ, USA.; ^3^The Hastings Center, Garrison, NY, USA.

**Keywords:** Aging, Pensions, Healthcare Spending, Defining Long-term Care, Public and Private Responsibilities, International Comparison

## Abstract

Japan has been aging faster than other industrialized nations, and its experience offers useful lessons to others. Japan has been willing to expand its welfare state with a long-term care (LTC) insurance to finance home care and nursing home care for frail elderly. As Ikegami shows, it created new facilities and expanded specialized staffing for home care, developed a country-wide assessment system and shifted responsibilities between the central and local authorities over that assessment and the determination of co-payments for LTC. Faced with rapid growth in demand for LTC, the government felt the need for new cost control measures. The Japanese experience illustrates that new social policies take time to develop. There is often a need to adjust. But there are also other lessons. The main one is that there is no direct relation between the degree of population aging and total health spending. While aging requires adjustments in the organization of care, and expanding LTC for frail elderly, international studies show there is no need to worry about the ‘unaffordability’ of aging. In this commentary, we have framed four "What, Why, Who, and How" questions about LTC to (re-)define the borderlines between public and private responsibilities for the range of activities for which some (but certainly not all) frail elderly as well as many non-elderly require support in daily life.

## Introduction

Japan has been aging faster than any other industrialized nation. Its experience is therefore important to others: how can we adapt to the aging of our populations, especially in the fields of pensions and healthcare?

Brief, there are three possible policy directions. One is to counter that demographic process by trying to encourage families to have more babies to reverse the aging process (eg, as France has tried, by giving financial incentives, preferential access to public transport or education, and good childcare). The results of those policies have been mixed, to say the least, and do not seem to have turned around the trend of leveling off and even declining population size in many nations. It is also not clear whether the world need more population growth. The second policy direction is adjustment of public pension systems. German’s chancellor Bismarck set the threshold for old age pension for industrial workers in 1883 at 65 years. In 1883, policy-makers knew, these workers would live, on average, another 3 years after 65. Today, we live, on average, almost 30 years after that age but we still think of 65 as the retirement age (in some cases 67, but in several countries, well below that age). This demographic change obviously requires adjustments in pensions, for example, reversing trends of early retirement, freezing or even cutting pension payments and encouraging higher labor market participation.^1-4^ Many Organisation for Economic Co-operation and Development (OECD) (and other) countries have already taken such measures.

There is little or no relation, however, between population aging and the level of healthcare spending.^5-7^ Some nations with much older population than the United States, for example Japan, Germany, or the United Kingdom, spend much less on healthcare, often with better outcomes in terms of life expectancy or child mortality. The incomes of health professionals, the use of medical technology and the organization of healthcare services are all factors that have played a greater role in determining the level of health spending than aging.^8,9^ That is also true for the costs of end-of life care.^10,11^

A century ago, working people, especially the blue-collar beneficiaries of the German social insurance schemes, were generally worn out by the end of their working lives. Aging as a demographic process is no longer just a burden to society. There are several positive sides to aging.^12^ Nowadays the vast majority of people in their 60s and 70s have good health and live independently.^13,14^ Some studies show that the total value of transfers of the older generation to their children and grandchildren is larger than the net cost of (public) pensions and long-term care (LTC). Many of the currently retired support their offspring financially and materially in paying for education, lending money to buy an apartment or house, and providing childcare or transportation services to grandchildren (see Gusmano and Okma^7^). Depicting populations over 65 as ‘frail dependent elderly’ is at least misleading.

The need for support by family or external professionals generally only rises substantially after elderly reach 80 or 85 years, and even at that age, only for a minority. The number of dependent frail elderly is growing rapidly, but it still represents a modest share of the total population.

The third policy direction is therefore to accept the reality of aging populations and design policies that support this demographic change, including LTC. This brings us to our four “What, Why and Who, and How” questions. What is LTC; Why is it important to find an operational definition of the range of activities that fall under LTC; Who is involved; and How can we draw lessons from the experience of Japan (and from other OECD nations that have implemented LTC insurances)?

## 1. What

When becoming frail, elderly need more support by family and others, including long-term (medical, nursing, and social) care. Ikegami^15^ refers to the definition of the Institute of Medicine: “…. a variety of ongoing health and social services provided for individuals who need assistance on a continuing basis because of physical or mental disability. Services can be provided in an institution, the home, or community, and include informal services provided by professionals or agencies.” That the Institute of Medicine definition is not very operational, however. As Ikegami argues, we need to define better what is it we are discussing. There is no clear borderline between services ‘for the elderly’ and others. For example, we all start our lives 100% dependent as pretty helpless babies and that dependency can last for many years. Second, adults with mental or physical handicaps may need life-long external support in their daily living. (There are also healthy adults who prefer to contract out (if they can afford to do so) many of the daily activities that would fall under the above definition of ‘LTC’). Even while focusing on the needs of disabled and elderly population groups we still need to make the term LTC more operational.

## 2. Why

There are two important dimensions to LTC: time and scope. The time dimension means that in practice there is often a gradual expansion of the scope of LTC. For example, elderly who still live by themselves do need some help, first with filling in taxes or walking their dog, for example, then with gardening. The next stage may be some shopping and housekeeping. These are all activities often done by close relatives or neighbors, mostly spouses and children. Then, there may be increased need for daily personal care, like preparing food and dressing. That stage becomes more intimate with assistance in eating, showering, helping to go to the toilet and preparing to go to bed. It also overlaps with nursing care to preventing bed sores, wound care, taking medicines, and keeping contact with other medical care when needed, eg, in refilling prescriptions, referral and after hospital care. We thus take a wider perspective of LTC than Ikegami who mostly focuses on professional medical and nursing care.

The gradual increase of frailty or sometimes sudden onset of dependency, for example after hospitalization, often leads to a situation where spouses or children and other family members find themselves in care roles they had not really expected or planned for. Acknowledging the two dimensions of LTC requires planning ahead and thinking about future shifts in activities and responsibilities.

## 3. Who

Family members responsible for the LTC of the parents, grandparents or other aging relatives face important decisions during all those stages, often implicitly. Are spouses and children willing and able to face the growing burden of LTC? At what stage do they formally or de facto take over the decision-making power of their frail parents? How can they respect the preferences of the elderly themselves? Who is responsible for the finances? Is there enough money to hire external help for housekeeping and other daily activity (or long-term stay in a nursing facility)? What range of services is covered by public or private insurance? What are the financial conditions? Who is responsible for the copays and services not covered by insurance? Are there time limits of the coverage? Who is responsible for the coordination between the formal and informal services?

Answering these questions requires a political debate about the shifting responsibilities between the private and the public domains. All industrialized nations have expanded public financing of LTC for elderly and other populations. At the same time, the largest part of LTC is still provided by spouses, children, neighbors and other informal caregivers (some studies estimate that up to 90% of all LTC still takes place within the family). In some cases, as in Japan, Germany and the Netherlands, informal care givers can receive financial compensation financed out of the cash benefit schemes. In parallel, there has been expansion of LTC services by (both for profit and non-profit) agencies. While there has been some growing acknowledgement of the principle of public (state) responsibility over LTC financing across nations, there is wide variety in the actual provision and organization of those services. In all cases, LTC involves many different stakeholders.

## 4. How

As noted above, most people over 65 live independently and consider themselves in good health (see also Gusmano and Okma^7^). We argue that redefining ‘aging’ for the purpose of LTC takes much of the sting out of the ‘tsunami of aging’ debate (for this argument see Gusmano and Okma^7^). A new, more realistic definition of aging as the population share of over, say, 80 will show that we face demographic change, but the consequences are not as dramatic as many policy-makers or politicians have assumed or feared. Moreover, demographic change is slow, so we have decades to adjust. So what adjustments?

The first are fiscal ones. As mentioned above, the major extension of average life expectancy in the last century means that we have to adjust our pension systems. We have to make sure that the public pension systems include as many people as possible, as long as possible, joining early in life and contributing all trough working lives. Moreover, there is no reason to encourage early retirement or full retirement benefits without a minimum of, say, 35 to 40 years of contribution. The systems should also become more flexible in including part time workers and providing credits for taking time off to take care for very small children and frail parents or relatives. Importantly, the pension benefits should be high enough to cover a decent minimum cost of living (in the United States, over 60% of retirees depend on Social Security as the main income source^16^). At the same time, there is no reason to generally exclude elderly citizens from fiscal arrangements. The current generation of elderly is, on average, not poor. Many have accumulated investments, pension savings and other wealth and there is no reason to exclude those from taxing merely because of age.

That situation is different for healthcare and LTC, however. The vast majority of retired US citizens have Medicare healthcare insurance. Still, many face high copayments and deductibles. Some studies estimate that in the US elderly over 85 spent about US$24 000 out of pocket for LTC in 2017. With a Social Security income of about US$1400 per month, such costs are obviously not affordable for elderly without substantial additional pension incomes and savings.

After defining LTC as the range of activities above for which frail elderly (and others) need assistance from relatives and or others, we thus have to (re-)establish borderlines between public and private (family) responsibilities for incomes, housing and other daily needs. The good news is that in general, the need for LTC only kicks in at a later age—and only for a smaller share of the population over 80. We can plan for that, fiscally, organizationally, and in regard to manpower.^17^ There are many examples across the world of countries that have already experimented with models of LTC. Ikegami’s contribution illustrates how Japan introduced and adapted several plans and policies for its aging population. These included the introduction of new LTC insurances, first the reduction but later increases of coinsurance, the redefinition of the role of hospitals and new LTC facilities, a country-wide assessment tool for home care, training of home helpers and expansion of day care facilities for elderly, shifts in the responsibilities over needs assessment and user fees between the national and local level. The development of LTC policies also entailed several attempts of cost containments like reduced fees for providers, cutting benefits and means testing (as in other countries, the latter measures were only moderately successful).

Japan’s experience is not unique. Holland was the first country that introduced its population-wide LTC insurance (the Exceptional Medical Expenses Act, AWBZ, of 1968). The AWBZ aimed to alleviate families and private charities from the financial burden of taking care of frail elderly and handicapped relatives. That fueled a rapid expansion of the capacity of retirement homes, nursing homes and other care institutions. It also allowed the existing (private but non for profit) home care organizations to expand their services. Three decades later, in an effort to rein in public LTC spending (in particular the popular cash benefit scheme) and to reduce the volume of institutional care, Dutch government basically dismantled this LTC insurance and largely shifted responsibilities to the municipal level.^18^ Germany followed the Dutch model and introduced its own LTC insurance in 1995. This social insurance mostly benefitted the existing welfare agencies that already were providing both home care and inpatient services. It also included a cash benefit scheme for families providing care for their frail elderly. The introduction of the LTC arrangements, in all three nations, expanded the public responsibilities for the financing and provision of LTC. While the organizational form and administrative responsibilities have shifted over time, in all three nations these arrangements became firmly embedded as integral part of the modern welfare states, fueling public expectation that the state would take responsibility for LTC for the elderly.

## Ethical issues

Not applicable.

## Competing interests

Authors declare that they have no competing interests.

## Authors’ contributions

Both authors worked on conceptualization, writing, and editing of the manuscript.

## Authors’ affiliations

^1^Independent Researcher. ^2^School of Public Health, Rutgers University, New Brunswick, NJ, USA. ^3^The Hastings Center, Garrison, NY, USA.
